# Novel anti-glioblastoma agents and therapeutic combinations identified from a collection of FDA approved drugs

**DOI:** 10.1186/1479-5876-12-13

**Published:** 2014-01-17

**Authors:** Pengfei Jiang, Rajesh Mukthavavam, Ying Chao, Ila Sri Bharati, Valentina Fogal, Sandra Pastorino, Xiuli Cong, Natsuko Nomura, Matt Gallagher, Taher Abbasi, Shireen Vali, Sandeep C Pingle, Milan Makale, Santosh Kesari

**Affiliations:** 1 Translational Neuro-Oncology Laboratories, Moores Cancer Center, UC San Diego, La Jolla, CA 92093, USA; 2 Department of Neurosciences, UC San Diego, La Jolla, CA 92093, USA; 3 Moores Cancer Center, UC San Diego, 3855 Health Sciences Drive, La Jolla, CA 92093, USA; 4 CellWorks Inc., Irvine, CA USA

**Keywords:** Glioblastoma, Drug screening, Patient-derived glioblastoma cell lines, Rational combination

## Abstract

**Background:**

Glioblastoma (GBM) is a therapeutic challenge, associated with high mortality. More effective GBM therapeutic options are urgently needed. Hence, we screened a large multi-class drug panel comprising the NIH clinical collection (NCC) that includes 446 FDA-approved drugs, with the goal of identifying new GBM therapeutics for rapid entry into clinical trials for GBM.

**Methods:**

Screens using human GBM cell lines revealed 22 drugs with potent anti-GBM activity, including serotonergic blockers, cholesterol-lowering agents (statins), antineoplastics, anti-infective, anti-inflammatories, and hormonal modulators. We tested the 8 most potent drugs using patient-derived GBM cancer stem cell-like lines. Notably, the statins were active *in vitro*; they inhibited GBM cell proliferation and induced cellular autophagy. Moreover, the statins enhanced, by 40-70 fold, the pro-apoptotic activity of irinotecan, a topoisomerase 1 inhibitor currently used to treat a variety of cancers including GBM. Our data suggest that the mechanism of action of statins was prevention of multi-drug resistance protein MDR-1 glycosylation. This drug combination was synergistic in inhibiting tumor growth *in vivo*. Compared to animals treated with high dose irinotecan, the drug combination showed significantly less toxicity.

**Results:**

Our data identifies a novel combination from among FDA-approved drugs. In addition, this combination is safer and well tolerated compared to single agent irinotecan.

**Conclusions:**

Our study newly identifies several FDA-approved compounds that may potentially be useful in GBM treatment. Our findings provide the basis for the rational combination of statins and topoisomerase inhibitors in GBM.

## Introduction

Novel therapeutic options are sorely needed to target glioblastoma (GBM), a notoriously treatment-resistant brain cancer. GBM is a leading cause of cancer-related death in the pediatric and adult populations, with most patients succumbing within 1-2 years [1,2]. The standard therapies are inadequate, and their toxicities lead to severe life-long morbidity in the small number of patients that survive [2]. Despite this grim prognosis, GBM is an orphan disease that is in general not a priority for new drug development [3-5]. Moreover, the biology of GBM is complex and much remains to be learned about the putative key signaling pathways before they can be therapeutically exploited [6]. In view of the unmet and urgent clinical need, we were motivated to pursue recent data indicating that GBM may respond to some FDA-approved agents not previously identified as GBM therapeutics [7,8]. The *in vitro* screening of a broad range of drugs already approved for other indications is attractive as *in vivo* toxicity and pharmacology are well defined, and such compounds can enter GBM clinical trials rapidly either as single agents or as combinations.

Accordingly, our goals were to identify and characterize single and combination agents having anti-GBM activity that we can potentially introduce into clinical trials quickly. To this end, using GBM cell lines and patient-derived GBM cell cultures, we screened a 446-compound NIH Clinical Collection (NCC) library comprising FDA-approved drugs. To further improve the anti-GBM potency of these drugs, we tested various drug combinations and compared them to single drug treatment.

Our screening strategy included multiple human GBM cell lines of different origins in order to provide cross-validation of findings. These cell lines included 4 established serum-grown, immortalized human GBM lines, 4 patient-derived stem cell like GBM primary cells grown as neurospheres, and 2 primary patient-derived GBM lines grown as adherent cultures [9-14].

We did not confine our screening to only adherent GBM stem cell lines despite reports claiming that such lines remain undifferentiated longer and constitute a simpler, less variable assay [11,14]. It is not yet firmly established that the major therapeutic target in GBM is just the cancer stem cell tumor compartment and there are indications that other cell types within GBM may assume stem cell characteristics through genetic or epigenetic events [14-16]. In contrast to a single type or lineage of cells, neurospheres contain a mix of GBM stem cells and differentiated cells, which is more reflective of the composition of human GBM tumors [17]. Further, when dissociated neurospheres are implanted orthotopically, they are highly tumorigenic and authentically recapitulate the invasive natural history, composition, and histology of GBMs growing in humans [16]. Hence we report the identification of NCC active compounds through our screening approach on patient-derived stem cell-like GBM primary cells.

Our initial screening identified 22 compounds active against GBM (>50% cell death) [18] at pharmacological doses. These 22 compounds encompassed 11 drug classes. In particular, we found that the statin, pitavastatin, effectively induced cellular autophagy and suppressed tumor cell MDR-1 protein, to impressively enhance the potency of irinotecan, a topoisomerase 1 inhibitor used in cancer treatment [19-21]. This work newly identifies FDA approved drugs and drug combinations, notably pitavastatin and irinotecan, that may be useful for GBM treatment, and draws attention to the potential value of *in vitro* screening of approved compounds not currently used to treat GBM.

## Materials and methods

### Overview of cell-based *(in vitro)* screening for potential anti-GBM compounds

We acquired 446 small molecules that completed human clinical trials from the NIH Clinical Collection (NCC). The initial broad screen was performed on U87 cells plated at 2000 cell per well on 96-well plates incubated overnight. All compounds were added to the plates to attain a final concentration of 10 μM. After 72 hours of incubation with drugs, the inhibition of cell proliferation was quantified by the Alamar Blue viability assay. Briefly, after incubation, Alamar Blue (#BUF012B, AbD Serotec) was added directly to the culture medium, and the fluorescence measured at 560/90 to determine the number of viable cells (Infinite M200, Tecan Group Ltd.). The IC_50_ values were calculated using commercially available software (Prism®, Graphpad Software, La Jolla, CA). We defined active compounds as those eliciting a greater than 50% reduction of cell viability in three independent screens. The 15 most potent and available drugs or compounds were then re-screened with other established glioma cell lines (LN443, A172 and U118), with the four patient derived GBM stem cell like primary neurosphere lines (SK72, SK262, SK429 and SK660), and with 2 GBM stem cell like primary cells (SK72 and SK262) grown as adherent culture. Pitavastatin was also tested in combinations with the other 12 compounds. The IC_50_ values were determined with and without pitavastatin (2 μM), using the Alamar blue assay as described above.

### Isolation, culture, and compound activity testing with patient derived GBM cells

#### Human GBM samples

Fresh human GBM material was acquired from 4 GBM surgical patients and cultured as previously reported [9,13]. Briefly, the dissociated tissue was washed, filtered through a 30 μm mesh and plated onto ultra-low adherence flasks at a concentration of 500,000 to 1,500,000 viable cells/ml. The stem cell isolation medium included human recombinant EGF (20 ng/ml), human bFGF (10 ng/ml) and heparin (2 μg/ml). Sphere cultures were then passaged by dissociation, washed, resuspended in neural stem cell culture medium (#05750,Stemcell Technologies), and plated on ultra low-adherence 96 well plates at 2000 cells per well for all subsequent drug testing.

Alternatively, patient-derived dissociated GBM tissues were plated onto laminin-1 coated plates (Sigma, 3-5 μg/ml). Cell populations were dissociated using Acutase (Sigma) and expanded for 5-10 passages, then plated on flat bottom for drug testing.

#### Confirmation of stem cell marker expression

Primary neurospheres were cytospun onto glass slides. Adherent primary cultures were grown onto Permanox™ chamber slides (#70380,EMS). Cells were incubated with human Nestin antibody (M1259, R&D system) and then with fluorescein-labeled secondary antibodies, then stained with DAPI. The cells were visualized under a UV microscope (Olympus BX51).

#### Drug testing and survival assay

As explained above, cells were seeded onto either regular or ultra-low adherence 96 well plates and incubated for 18-24 hours and then treated with vehicle control or single drugs or drug combinations. After 96 hours of incubation, Alamar Blue was added directly to the culture medium, and the fluorescence measured at 560/90 after 4-12 hours to determine the number of viable cells. The IC_50_ was calculated.

### Prediction of blood brain barrier (BBB) permeation by active compounds

Although ample evidence has demonstrated that drugs of virtually any size or chemotype can enter brain tumor via leaky tumor microvessels, the ability to penetrate the intact blood brain barrier (BBB) is reasonably hypothesized to be useful for treating tumor cells infiltrating normal brain tissue along fiber tracts [22-25]. Hence we estimated the capacity of active anti-GBM compounds to cross the BBB. We used standard software to calculate the Log BB value: Log BB = -0.0148 PSA + 0.152 CLogP + 0.139; PSA = polar surface area, p = octanol/water partition coefficient [26,27].

### Determination of cell cycle, autophagy, and apoptosis

#### Cell cycle analysis

GBM cells were seeded into 10 cm dishes at a density of 1 × 10^6^, cultured overnight followed by the addition of 3 μM pitavastatin with 24 or 48 hours of incubation. Cells were trypsinized and fixed in 70% ethanol for 30 minutes, incubated with 25 μg/ml propidium iodide (PI) and 250 μg/ml RNAase in PBS for 1 hour at 50°C. After PI staining, cells were analyzed via flow cytometry (Canto, BD FACS), and the percentage of cells in G_0_/G_1_, S and G_2_ phases were calculated by ModFit LT software version 3.0.

#### Detection of caspase activity

Caspase-3 activity was measured with the Invitrogen Enzcheck caspase-3 assay kit #2 according to the manufacture’s protocol. Briefly, 3 × 10^6^ U118 cell were cultured and pitavastatin, irinotecan or the combination was added to the medium for 12 or 24 hours. Then 10^6^ cells were lysed, DEVD-R110 fluorescence substrate was added, and the fluorescence signal was measured and compared with a standard curve (Infinite M200, Tecan). Caspase 3/7 activity was measured by the Apo-One caspase3/7 Kit (#G7790, Promega). 20,000 cells were seeded on to 24 well plates, pitavastatin and vehicle were added, followed by incubation (8, 12, 24 and 48 hours) and caspase 3/7 activity was measured using a fluorescence-based substrate.

#### Detection of autophagy markers-GFPLC3 punctuation

Retrovirus carrying the GFPLC3 was produced by transfecting the 293GP2 cells with the pVSV-G and pBABE-puro-GFPLC3 plasmids (GFP fused with LC3 gene at the N-terminal, Addgene #22405). Retroviral supernatants were harvested 48 hours later. U87, U118, U251 cells were seeded at a density of 2 × 10^5^ in 6 well plates and infected 24 hr later with the VSV-G/GFPLC3 virus. Stable cell lines were selected for 1 week in 1 μg/ml puromycin. GFPLC3-expressing lines were seeded onto 24 well plates and treated with 1 μM pitavastatin for 48 hours. Presence of GFPLC3 punctuation, which is a marker of autophagy was detected by UV microscopy (Olympus IX81).

#### Western blot analysis for autophagy, apoptosis, and multidrug resistance protein

LC3 (autophagy), caspase-3 (apoptosis), and MDR-1 (multidrug resistance protein 1 or P-glycoprotein 1,ABCB1) and tubulin (as loading control) were detected by western blotting following drug treatment. Cell lysates (10-50 μg protein) were loaded on to either 14% SDS-PAGE gel (for LC3) or 4-12% gel (for caspase-3, and MDR-1), proteins transferred to PVDF membrane and probed with primary antibodies (L8918 Sigma, 9662 Cell signaling, Sc-8313 Santa Cruz, T4026 Sigma). The resultant protein bands were visualized by a supersignal kit (#1856136,Thermo Scientific) after incubation with HRP-labeled secondary antibodies.

#### Multi-drug resistance assay

A cell-based fluorescence assay kit (Cayman Chemical Company, MI, USA, #600370) was used to evaluate modulation of the MDR-1 protein by drugs. Calcein AM is a hydrophobic non-fluorescent dye that easily permeates living cells. The hydrolysis of Calcein AM by intracellular esterases produces calcein, a hydrophilic strongly fluorescent compound which is retained in the cell cytoplasm and can be measured using excitation and emission wavelengths at 485 nm and 535 nm, respectively. Calcein AM is a substrate of MDR-1 protein P-gp, which causes its rapid extrusion from the plasma membrane, preventing accumulation of the fluorescent calcein inside the cytoplasm. Therefore measurement of fluorescent calcein allows for detection of MDR activity in live cells. Hoechst Dye staining of nuclei measured using of excitation and emission wavelengths 355 nm and 465 nm respectively to normalize cell numbers in well. GBM cells were seeded at 5 × 10^4^/well overnight, then pitavastatin was added to final concentration of 1, 3 and 10 μM. Twenty four hours after treatment, cells were incubated for Calcein AM/Hoechst Dye solution for 15 min, then fluorescent Calcein retention was measured 20 μM Verapamil or cyclosporine A treatments for 20-30 min as positive control of MDR-1 inhibition followed as the manufacturer’s protocol. The results were expressed as ratio of Calcein AM/ Hoechst signal (C/H ratio). Photomicrographs were taken using fluorescence microscopy.

#### GBM patients’ survival and free disease status relative to MDR-1 (ABCB1) expression

The GBM patient data were obtained from The Cancer Genome Atlas (TCGA) public data portal, and analyzed using the cBio Cancer Genomics Portal. This system is developed and maintained by the computational biology center of Memorial Sloan-Kettering Cancer Center. We investigated and regrouped GBM patients according their MDR-1 (ABCB1) expression. Firstly, we required the patients/case ID with the MDR-1 expression in all TCGA GBM provisional databases. The mRNA expression z-scores threshold were set as ±1 in our analysis. Then, we regrouped all the patients into 3 groups according to their MDR-1 expression as up-regulated, normal, and down-regulated. Finally, we inputted the down-regulated or up-regulated patients ID with normal expressed patients to select patient/case set to analyze patient survival and free disease status data. The Kaplan-Meier curves were drawn based on these analyses.

#### Animal studies

The *in vivo* studies were performed on nude mice to evaluate the drug effects on inhibition of tumor growth. 2 × 10^6^ U87 cells were subcutaneously transplanted into the right and left flanks. Initial tumor growth was monitored every 3 days. Drug administration was initiated when the tumors reached a size of 100-120 mm^3^. Mice were regrouped into 5 groups of 6 mice each, without significant difference in tumor volume before drug treatment. The mice were treated with either PBS as control, low dose of pitavastatin (0.5 mg/kg), low dose of irinotecan (0.5 mg/kg), a combination of pitavastatin (0.5 mg/kg) and irinotecan (0.5 mg/kg), or high dose of irinotecan (5 mg/kg). All drugs were injected i.p. in 200 μl of PBS, once per day, on a 5-days-on, 2-days-off schedule. Tumors size and mice weight were measured 2 times per week. All mice were sacrificed after tumor sizes reach over 1 cm in diameter in the control group. Tumor volumes were calculated as (length*width*width/2). After sacrifice, all tumors were disserted and weighted. The animal protocol was approved by UCSD Institutional Animal Care and Use Committee (IACUC).

#### Statistical analysis

Activity against GBM cells was assessed by dividing the average number of viable cells (from three replicates per dose) by the average of three controls. At a type I error rate of 0.05, using a one-sided t-test, we calculated 80% power to evaluate whether a decrease in mean percent viable cells was significantly lower than 100%, if the observed mean percentage was 91.4%; we conservatively assumed the standard deviation of the percent viable cells was 15%. For significant difference by t-test (P < 0.05), labeled * at the bar graphs.

To quantify the synergism of drug combinations, the drug combination index (CI) was calculated as described by Chou [28]. ED_50_, ED_75_ and ED_90_ were defined as the drug dose able to inhibit cell growth 50%, 75% and 90%, respectively, for pitavastatin alone, irinotecan alone and mixture of two drugs (various ratios). A CI < 1 indicates synergy between the two drugs.

## Results

### *In vitro* screening of drugs

#### U87 studies

**Figure 1:**
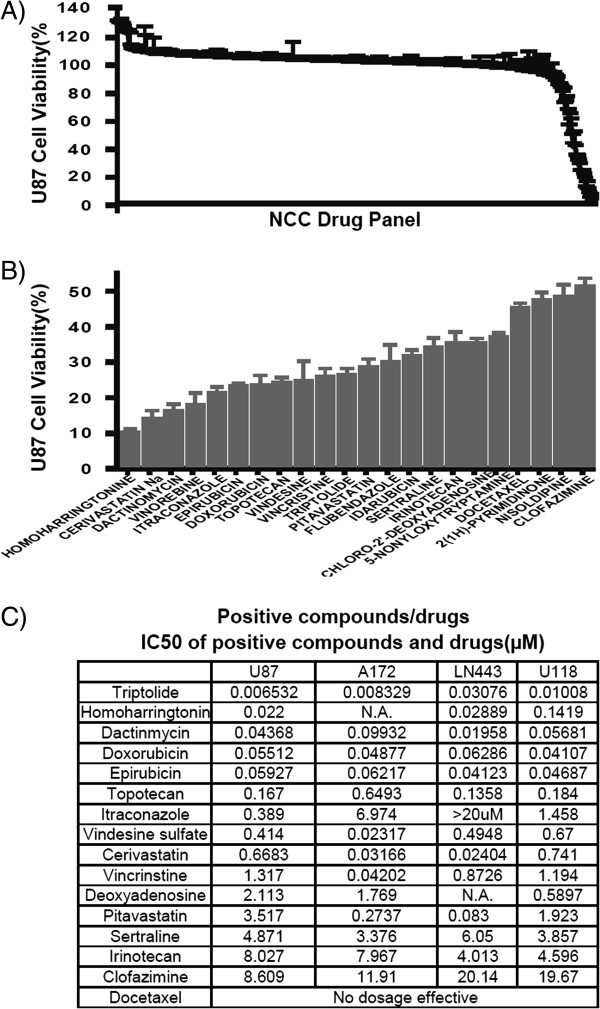
**NIH clinical collection compounds/drugs screened *in vitro *with U87 human GBM cells. (A)** All 446 compounds in the NCC drug panel ordered according to percent viability after treatment, note the shoulder at the end indicating 22 agents with anti-GBM activity. **(B)** The 22 positive compounds retested with three replicates. **(C)** IC_50_ of the most active and available compounds screened with four widely used, established GBM cell lines. The predicted BBB value for each compound is shown.

The U87 *in vitro* cell culture platform was used to initially screen the NCC library of 446 small molecules. We calculated percent cell viability as depicted in Figure 1A, and found that 22 drugs reduced viability to less than 50%. Figure 1B shows the specific cell viability for each of these 22 compounds. Homoharringtonine and cerivastatin reduced survival to 10% percent or less, while 9 compounds reduced survival to less than 25%, 6 drugs reduced survival to less than 35%, and the remainder was associated with a survival of 35-50%. As single agents, all these 22 compounds are more effective *in vitro* than temozolomide, a widely used drug in GBM treatment (Additional file 1). This result is consistent with previous studies [29,30]. Nine of the 22 compounds producing <50% cell survival were more potent than vincristine, a component of a commonly used glioblastoma chemotherapy regimen (procarbazine, lomustine and vincristine – PCV). Similarly, 15 of the 22 compounds were more potent that the commonly used GBM chemotherapeutic irinotecan. As expected, most of the compounds (13 of 22) were antineoplastics and a majority of these (8) oncology drugs are not currently used for the treatment of GBM. Three cardiovascular compounds, cerivastatin, pitavastatin, and nisoldipine showed activity, with the two cholesterol-lowering agents, cerivastatin and pitavastatin having the greatest effect. The effectiveness of statins prompted us to test a range of commercial available statins; of which, cerivastatin and pitavastatin have the lowest IC_50_ values (unpublished data).

The two serotonergic pathway inhibitors, sertraline and 5-nonyloxytryptamine also inhibited the survival of U87 cells, which agrees with previously published findings using an adherent GBM stem cell assay [11,12].

#### A172, LN443 and U118 cells

To further characterize the most potent compounds identified in our initial screen, we re-screened, using the established cell lines A172, LN443, and U118, the 15 compounds that showed the highest potency with U87 cells (Figure 1C). We found that 8 drugs had greater potency than vincristine in all cell lines tested and 12 drugs had lower IC_50_ values than irinotecan. We selected 8 FDA approved drugs for further investigation using patient-derived GBM stem cell-like cells (D-Actinomycin, Liposome-encapsulated Doxorubicin/Doxil, Epirubicin, Irinotecan, Pitavastatin, Sertraline, Topotecan and Vincristine).

#### Stem cell-like GBM lines

**Figure 2:**
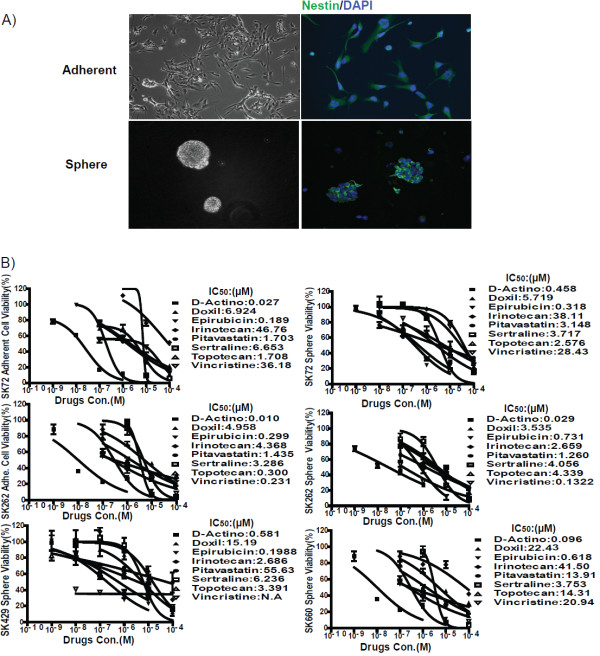
**Drug potency against stem cell-like GBM neurosphere derived from patient surgical isolates. (A)** Photomicrographs of primary cells growing as adherent culture and neurospheres and expressing the neural stem cell marker Nestin. **(B)** Eight FDA approved drugs showing best activity in established cell line screens tested in duplicate with adherent cultures and with neurospheres. In each case D-actinomycin exhibited the lowest IC_50_.

We used GBM stem-like cells derived from surgically-resected patient samples. Previously, using whole-exome sequencing, we observed global conservation of the patient’s tumor genetics in various pre-clinical models, including neurospheres, adherent cells and xenografts [31]. Findings from our study therefore support the use of GBM stem-like cells for the development and testing of personalized targeted therapies. In the present study, we used GBM samples from 4 patients that formed neurospheres in culture. Two of these cell lines (SK72 and SK262) also formed adherent cultures. We found that both the neurospheres and adherent cultures expressed equal and high levels of the neural stem cell marker Nestin. Figure 2A shows photomicrographs representative of Nestin staining performed on SK72 neurospheres and SK72 adherent culture. All 8 FDA approved drugs with activity against U87 cells also had IC_50_ values lower than two currently used anti-GBM agents, vincrinstine and irinotecan in GBM stem-like cells (neurospheres and adherent cultures). D-actinomycin and epirubicin exhibited the greatest potency (Figure 2B), and the liposomal form of Doxorubicin (Doxil) was less potent than epirubicin even though their IC_50_ values with U87 cells were virtually the same (data not shown).

The topoisomerase 1B (Topo 1) inhibitor topotecan exhibited potency that significantly surpassed the structurally related Topo 1 inhibitor irinotecan. Similarly, two statins exhibited good activity, which is promising as these drugs have low toxicity and owing to their target pathways may enhance the activity of currently used oncologic agents via synergism. The IC_50_ for pitavastatin was less than 10 μM in most of our cells tested (a range of 1.260 to 55.63 μM). Similarly, the IC_50_ of sertraline was in the range of 3.1 to 6.6 μM.

#### Predicted blood brain barrier permeation values of pitavastatin

The ability of pitavastatin to cross the BBB is predicted to be limited as the –log BB was calculated as -0.6499. However, the drug circulates freely in plasma and may enter the enhancing component of tumors via permeation through typically leaky tumor microvessels [25,32].

### Effect of pitavastatin on GBM cells

Considering the effectiveness of statins in our study, specifically pitavastatin in inducing cell death and owing to relatively fewer adverse effects, we decided to explore pitavastatin in detail.

#### Pitavastatin induces autophagy in GBM cells

**Figure 3:**
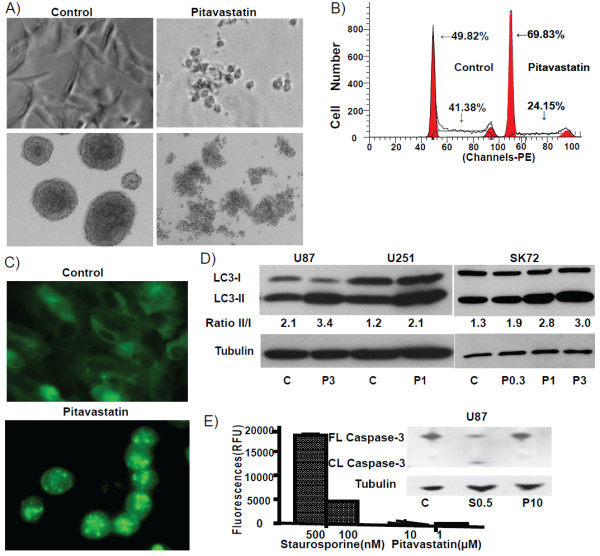
**Pitavastatin induces autophagy associated cell death for GBM. (A)** Pitavastatin induced SK72 glioma primary cell death in adherent and neurosphere cultures. Note rounding of cells at 24 h. **(B)** FACS analysis showing that pitavastatin treated U87 cells were arrested in G_0_/G_1_ phase with a much lower percentage of the total population in S phase. **(C)** In GFP-LC3 transduced-U87 GBM cells, pitavastatin induced a punctuate GFP-LC3 distribution, which is a characteristic of autophagy. **(D)** Western blot showing that pitavastatin induced the transformation of LC3 protein from isoform I to II in U87, U251 and SK72 cells. (Pitavastatin concentrations in μM). LC3I/II was calculated with NIH ImageJ software **(E)** Caspase-3/7 activity in U87 cells induced by staurosporine for 8 hours detected by the Apo-One fluorescence kit. In contrast, no caspase 3/7 activity was seen with pitavastatin treatment during 2 days. Inset: Western blot illustrating that there was no detectable cleavage (CL) of caspase-3 after pitavastatin treatment (P, 10 μM); Staurosporine treatment (S, 0.5 μM) was used as a positive control and was associated with caspase-3 cleavage (CL).

Pitavastatin induced cell morphologic changes, as early as 24 hours, with adherent cells assuming a rounded configuration and detaching from the substrate (Figure 3A). Death of tumor neurospheres was also triggered and these cells arrested in the G_0_/G_1_ phase after treatment (Figure 3B). G_0_/G_1_ phase cells were dominant and the proportion of cells in S phase dramatically decreased.

We found that pitavastatin-treated GBM cells exhibited characteristics consistent with autophagy rather than apoptosis [20,21]. After pitavastatin treatment, GBM cells showed extensive vacuolization, a key feature of cellular macroautophagy [33,34]. Further, pitavastatin-treated cells stably expressing the GFP-LC3 fusion protein developed a punctate cytoplasmic pattern, suggesting that GFP-LC3 covalently linked to phosphatidylethanolamine (PE) and was inserted into double membrane autophagosomes (Figure 3C).

Morphological observations were confirmed by Western-blot analysis of LC3, which revealed a LC3-I to LC3-II transition, a hallmark of autophagy (Figure 3D) [34,35]. The adherent versus sphere culture conditions did not influence the LC3 transition, which was observed in both U87, U251 adherent stable lines and in the stem cell-like SK72 cell spheres upon pitavastatin treatment. Furthermore, increasing concentrations of pitavastatin enhanced LC3-I to II transition (Figure 3D, right panel). In addition, Annexin staining failed to detect apoptosis after pitavastatin (data not shown) treatment. Caspase-3/7 activity was not detectable via fluorescence or by Western-blot analysis (Figure 3E). We could not entirely exclude the possibility that pitavastatin induced cell apoptosis by caspase independent pathways; however the cell cycle analysis shown in Figure 3B argued against this hypothesis, as it did not reveal a sub-G1 population, characteristic of apoptotic cells. The mechanism(s) of cell death induced by pitavastatin still needs more detailed investigation. Further, whether other statins can also trigger autophagy in human GBM cells remains to be determined, and this may depend, in part, on whether adherent cells or neurosphere cultures are assayed.

To elucidate the possible mechanisms by which pitavastatin decreases cell survival, we also used a virtual tumor cell technology. This is an *in silico* analysis using a comprehensive and dynamic representation of signaling and metabolic pathways underlying tumor physiology (based on CellWorks Inc. technology, Additional file 2). Using this platform, we tested the effect of pitavastatin on two GBM cell lines (A172 and U251) using genomic profiles. *In silico* modeling data predicted a significantly increase in autophagy makers in both GBM cells following pitavastatin treatment (Additional file 3).

### Drug combinations

**Figure 4:**
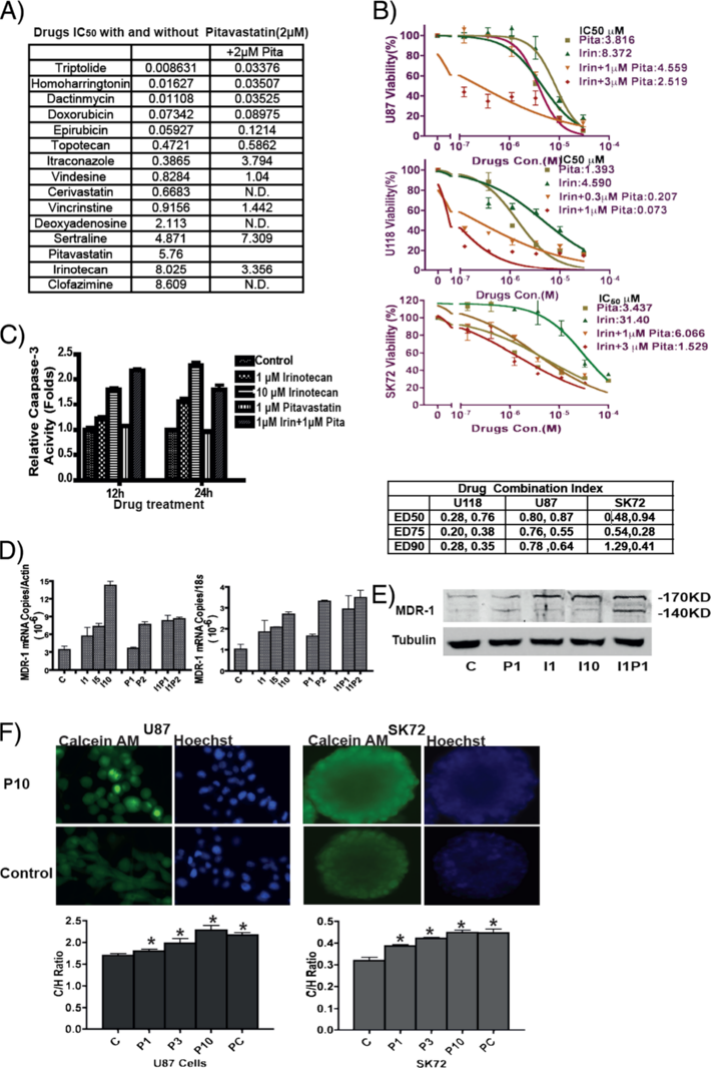
**Pitavastatin and irinotecan exhibit synergism in terms of inducing GBM cell death *in vitro*. (A)** U87 IC_50_ values for various compounds with and without pitavastatin (2 μM), note that irinotecan IC_50_ was reduced by 50%. **(B)** Pitavastatin, sharply lowered the IC_50_ of irinotecan in U118, U87 cells and SK72. The combination index calculated at ED_50_, ED_75_ and ED_90_. Data were calculated at two pitavastatin concentrations. **(C)** Escalation of apoptosis by addition of pitavastatin to irinotecan, particularly after 12 hours of incubation. **(D)** Pitavastatin and irinotecan applied singly induce MDR-1 mRNA transcription as shown by qRT-PCR, and when applied together the level of transcription is only slightly greater than with irinotecan alone. **(E)** Western blot analysis indicating that after irinotecan treatment, U87 expressed full length MDR-1 (170 KD), but in combination with pitavastatin, the band density of unglycosylated MDR-1 (140 KD) increased dramatically. Unglycosylated MDR-1 cannot be transported inside the cell and is essentially non-functional. **(F)** After pitavastatin treatment, U87 cell and SK72 cells were accumulated more Calcein AM and showed higher fluorescent signal than control by inhibited the MDR-1 function.

We then tested 12 drugs along with pitavastatin to investigate possible additive or synergistic effects. In these combinations tested using U87 cells, only irinotecan and pitavastatin displayed a synergistic effect, with effective lowering of IC_50_ for both compounds (Figure 4A). This synergistic effect was further confirmed in U118 and SK72 cells, using a concentration range of pitavastatin, which showed a dramatic 40-70 fold lowering of the IC_50_ compared to irinotecan alone (Figure 4B). Drug combination index (CI), calculated at ED_50_, ED_75_ and ED_90_, ranged from 0.28 -0.76 for U118 cells 0.55-0.87 for U87 cells and 0.41-1.29 for SK72 cells demonstrating a moderate-to-strong synergism between irinotecan and pitavastatin at various drug concentrations in all three GBM cell lines. Importantly, the addition of pitavastatin reversed the resistance of the primary SK72 neurosphere cells to irinotecan, causing a decrease in its IC_50_ from 30 μM to 1.5 μM.

### Enhancement of irinotecan via suppression of MDR-1 by pitavastatin

Irinotecan induces apoptosis, which is primarily responsible for its anti-tumor activity [36]. Although pitavastatin as a single agent did not induce apoptosis, in combination with irinotecan, it enhanced U87 caspase-3 activity as compared to irinotecan alone, both at 12 and 24 hours (Figure 4C). The major mechanism of drug resistance in GBM is the over-expression of the multi-drug resistance protein (MDR-1; p-glycoprotein 1 or ABCB1), seen in the BBB and neuroepithelial tumors such as GBM [37]. Multiple studies have established that MDR-1 is responsible for decreased drug accumulation in multidrug-resistant GBM cells [38,39]. Interestingly, pitavastatin is a substrate of MDR-1 [40]. We observed that MDR-1 gene transcription levels correlated directly with irinotecan concentration (Figure 4D). However, after combined pitavastatin and irinotecan treatment, the 140 KD MDR-1 band increased in intensity, suggesting MDR- glycosylation is suppressed, which attenuates the production of functional MDR-1 (Figure 4E) [41].

### Pitavastatin inhibited MDR-1 function

As shown in Figure 4D and E, pitavastatin induced MDR-1 mRNA and decreased glycosylation of MDR-1 protein. To elucidate the effect of pitavastatin on MDR-1 function, we evaluated the drug exclusion capability directly, using the Calcein-AM assay. As showed in Figure 4F, after statin treatment, both U87 and SK72 GBM cells showed increased intracellular amounts of the MDR-1 substrate (Calcein AM), indicating that pitavastatin may inhibit drug-exclusion mediated by MDR-1. The MDR-1 inhibition was directly proportional to pitavastatin concentration. This result suggests that the increased caspase activity, observed in cells treated with irinotecan in combination with pitavastatin, may be due to its MDR-1 inhibitory effects, which in turn caused accumulation of irinotecan (Figure 4C).

### Down-regulation of MDR-1 (ABCB1 gene) expression correlates with overall survival and longer disease-free status

**Figure 5:**
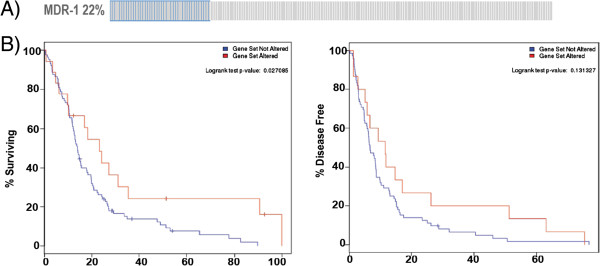
**Down-regulation of MDR-1 (gene ABCB1) predicted better survive for GBM patients. A)** Oncoprint mRNA expression map generated using cBio Cancer Genomics. 22% of a total of 243 GBM patients showed down-regulated MDR-1 (ABCB1) expression **B)** Down-regulation of MDR-1 (red-line) increases overall patient survival as the median survival (blue-line) increased from 14.1 months to 23.2 months in Kaplan-Meier curves (P < 0.05) and patients’ median progression-free survival increased from 6.67 to 11.54 months (P = 0.13).

In TCGA dataset, of the 243 GBM samples profiled, 43 showed down-regulation of MDR-1/ABCB1 (Figure 5A), 15 were amplified for MDR-1/ABCB1 and 34 had MDR-1/ABCB1 up-regulation. This result suggested that the MDR-1 transcription levels are variable and may be regulated by the tumor microenvironment. In all 173 cases with normal MDR-1 expression level (blue-line), the median survival was 14.1 months (n = 149) whereas in patients with MDR-1 down-regulation (red-line), it was increased to 23.2 months (P = 0.027; Figure 5B). Further, progression-free survival increased from 6.67 months in patients with normal MDR-1 to 11.54 months in case of MDR-1 down-regulation. For patients with MDR-1 up-regulation or gene amplification, there was no difference in overall or progression-free survival when compared to controls (data not shown). These data strongly suggest that MDR-1 inhibition following treatment with statins may have a beneficial effect in GBM patients.

### Combination of Pitavastatin and Irinotecan enhances anti-tumor efficacy *in vivo*

**Figure 6:**
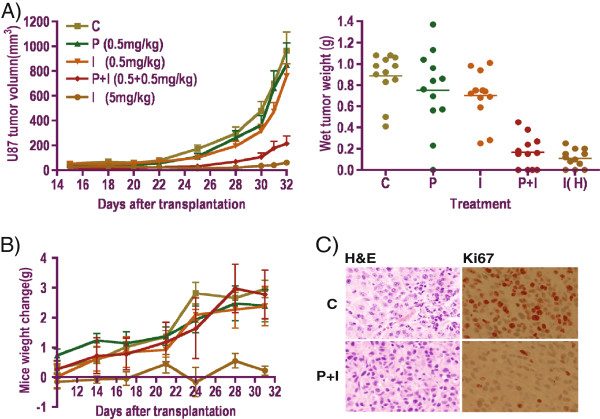
**Co-administration of low dose of pitavastatin and irinotecan inhibited tumor growth *in vivo*. A)** U87 tumor growth was inhibited by combination treatment but not by low dose of single drug; tumor volume and weight dramatically decreased following combination treatment when compared to control and single drug-treated groups. **B)** A combination of low dose pitavastatin and irinotecan showed no toxicity whereas high dose irinotecan induced mice body weight loss. **C)** H&E and Ki-67 staining of control and combination treatment of *in vivo* U87 tumor model, showed a much lower proliferation in the tumor cells after combination treatment.

To evaluate the *in vivo* anti-cancer effect of pitavastatin and irinotecan, we treated xenograft mouse models implanted with U87 cells with either single agent or combination. As shown in Figure 6A, low dose pitavastatin or irinotecan did not affect tumor growth. In contrast, 0.5 mg/kg pitavastatin in combination with 0.5 mg/kg irinotecan significantly attenuated tumor growth (P < 0.01) compared to both the control group and the low-dose single drug treatment groups (one week /5 drug treatment). Tumor measurements after sacrificing the mice at day 32 confirmed that combination treatment significantly reduced tumor size and weight (P < 0.001; Figure 6A, right panel). Interestingly, irinotecan administered as a single agent but at a dose 10-times higher than that used in the combination treatment group was also very potent in inhibiting *in vivo* U87 tumor growth. However, such high doses were associated with significant drug toxicity, as indicated by severe weight loss in drug-treated mice (Figure 6B). In contrast, the body weights of mice receiving a combination of pitavastatin and low-dose irinotecan increased 3-4 gram steadily similar to that seen in control and the low dose drug treatment groups during the whole study duration. Moreover, tumor cell proliferation decreased dramatically as showed by the Ki67 staining in Figure 6C.

## Discussion

In the present study, we sought to screen a library of FDA-approved compounds to rapidly identify new, non-GBM drugs that could be readily introduced into GBM clinical trials. Using a platform that employed a wide range of human GBM lines, including clinically relevant patient-derived primary GBM lines, our screening uncovered 22 compounds from different classes with anti-neoplastic activity in GBM. Among others, the cardiovascular drugs statins showed high efficacy in reducing tumor growth both *in vitro* and *in vivo*, drawing our attention to these relatively non-toxic cholesterol lowering drugs. The present study demonstrates the potency of pitavastatin relative to other statins. Importantly, our results demonstrated that co-administration of pitavastatin with low-dose chemotherapy, greatly increased the potency of the latter, lowering the IC_50_ values for irinotecan by 40- to 70-fold, with few adverse effects. Experimentally, we found that statins independently induced autophagy in GBM and that statins may potentiate chemotherapeutic agents by inhibiting MDR-1 function. This was consistent with *in silico* screening results using our virtual tumor cell technology, which suggested that pitavastatin affects cell viability by inducing autophagy.

Cholesterol has a key role in cell membranes, cell metabolism, cell signaling and has been implicated in tumor development and progression. Therefore, as cholesterol-lowering agents, questions about the anti-tumor effects of statins have been already posed [42,43]. Statins decrease cholesterol levels by inhibiting the enzyme HMG-CoA reductase in the liver. In addition, mevalonate, and isoprenoid intermediates such as geranylgeranylpyrophosphate (GGPP) and farnesylpyrophosphate (FPP) in the cholesterol synthesis pathway are also depleted after statin treatment [44]. Another intermediate, dolichol, an essential substrate for protein N-glycosylation, is also blocked by statins [45,46]. Considering that GBMs are highly proliferative taking up large quantities of cholesterol, potentially they may be vulnerable to statin treatment [47,48]. However, the mechanism of sensitivity of GBM to statins has not been elucidated. Recent studies have shown that statins may have an anti-GBM effect in xenograft mouse models, by targeting the low-density lipoprotein receptor (LDLR), inducing apoptosis via ERK/AKT pathway [20,47]. Other data hypothesize that statins may inhibit tumor growth by inducing autophagy via the NF-κB pathway in human colon cancer cell line [49]. Our data obtained in both stable cell lines and primary patient samples clearly demonstrated that pitavastatin induced macro-autophagy in GBM cells [20,21]. Further experiments are now ongoing to investigate the signaling pathway(s) involved in this effect.

Importantly, we have shown that pitavastatin potentiated the anti-tumor effects of low-dose irinotecan, a topoisomerase inhibitor. Pitavastatin is know to be a substrate of the multi-drug resistance protein, MDR-1, which is overexpressed in GBM upon drug treatment and is partly responsible for the resistance of GBM to chemotherapy. Our data indicate that, in combination with irinotecan, pitavastatin suppressed glycosylation of MDR-1, thereby inhibiting its function and allowing irinotecan to accumulate intracellularly [37,39,40]. Accumulation of irinotecan is likely responsible for the increased apoptosis in the presence of pitavastatin. The MDR-1 expression in cancer cells can be a significant obstacle to the success of chemotherapy. Many MDR-1 inhibitors have been extensively tested in clinical trials but the results have been inconclusive. According to TCGA data, down-regulated ABCB1 (MDR-1) predicted better survival of GBM patients. Combining a statin with a chemotherapeutic agent represents a powerful, potential strategy for circumventing resistance and significantly enhancing efficacy. Here we have confirmed that pitavastatin may improve the therapeutic response to TOPO-1 inhibitors, by inhibiting MDR-1 function, and may be beneficial for GBM patients. It remains to be determined whether other statins exert a similar or a different anti-neoplastic mechanism as compared to pitavastatin, and whether different subtypes of GBM have different sensitivity to pitavastatin or display other mechanisms for statin actions. GBM is a complex and heterogeneous disease that likely accounts for the different results obtained across various studies.

Irinotecan is broadly used in solid cancer therapy, especially in combination with other drugs [50,51]. In clinical use, the toxicity of irinotecan is generally manageable and reversible [52,53]. However, in some patients it may lead to severe side effects, such as diarrhea and neutropenia that can be life threatening. In our animal model, co-administration of pitavastatin allowed for a reduced dosage of irinotecan and avoided drug toxicity at higher dosage. These data indicate a new strategy to develop better irinotecan-based drug combination.

Based on the promising results of our present study, we are now undertaking additional preclinical studies of GBM to optimize dosing and characterize efficacy, thus providing a solid basis for a clinical trial with pitavastatin and irinotecan for the treatment of glioblastoma patients.

## Competing interests

The authors declare that they have no competing interests.

## Authors’ contributions

PFJ and SK designed the study and submitted paper. PFJ performed all experiments and collected all data. TA and SV performed in silico analysis. VF, SP, XC help analysis in vitro data. MG collected patients’ samples. RM, IBS, YC, NN helped on collected in vivo data. MM wrote the first draft of paper. SCP and SK revised the manuscript. All authors read and approved the final manuscript.

## Supplementary Material

Additional file 1: Figure S1 U87 cell viability after NCC drug treatment. U87 cell has various drug responses after single dose of all 446 NCC drug treatments. We showed all data in here which summarized in Figure 1A. The Top 22 hits were showed in Figure 1B. Click here for file

Additional file 2 ***In silico *** **modeling protocol.** Click here for file

Additional file 3: Figure S2 *In Silico* modeling predicts pitavastatin-induced autophagy in GBM cell lines. We simulated the effect of pitavastatin on the virtual tumor cell that modeled GBM cell lines A172 and U251. Our simulation demonstrated increased expression of autophagy and related pathway markers in both these cell lines. Click here for file
