# Correction: novel anti-glioblastoma agents and therapeutic combinations identified from a collection of FDA approved drugs

**DOI:** 10.1186/1479-5876-12-126

**Published:** 2014-05-13

**Authors:** Pengfei Jiang, Rajesh Mukthavaram, Ying Chao, Ila Sri Bharati, Valentina Fogal, Sandra Pastorino, Xiuli Cong, Natsuko Nomura, Matt Gallagher, Taher Abbasi, Shireen Vali, Sandeep C Pingle, Milan Makale, Santosh Kesari

**Affiliations:** 1 Translational Neuro-Oncology Laboratories, Moores Cancer Center, UC San Diego, La Jolla, CA 92093, USA; 2 Department of Neurosciences, UC San Diego, La Jolla, CA 92093, USA; 3 Moores Cancer Center, UC san Diego, 3855 Health Sciences Drive, La Jolla, CA 92093, USA; 4 CellWorks Inc, Irvine, CA, USA

## Correction

After publishing this article [1], we noted mistake in the spelling of co-author Rajesh Mukthavaram’s names. The corrected co author name has been included to the author list.
