# Supplementary material for: Novel anti-glioblastoma agents and therapeutic combinations identified from a collection of FDA approved drugs
Source: J Transl Med. 2014 Jan 17;12:13. doi: 10.1186/1479-5876-12-13 (PMC3898565; doi:10.1186/1479-5876-12-13)
Supplement: Additional file 2 — In silico modeling protocol. [file 1479-5876-12-13-S2.doc]

**SUPPLEMENTARY INFORMATION**

**Supplementary Methods**

***In Silico* Modeling**. We performed the simulation experiments and analyses using our virtual tumor cell technology, which is a comprehensive and dynamic representation of signaling and metabolic pathways underlying tumor physiology at biomarker level and converging on the key cancer phenotypes of proliferation, apoptosis, angiogenesis and metastasis. This platform provides a dynamic and transparent view of tumor cell physiology at the functional proteomics abstraction level. A bottom-up approach was adopted for building the complete dynamic system. Based on extensive literature search and development, all cellular processes comprehensively implicated in the pathophysiology of cancer were built as standalone modules. The modules were individually tested for correctness and predictive value by corroboration with experimental data and progressively integrated based on their crosstalk with other processes. The modeling of time-dependent changes in the fluxes of individual pathways required modified ordinary differential equations (ODE) and mass action kinetics. We have prospectively and retrospectively validated our simulation technology against extensive pre-defined in vitro and in vivo studies. The cancer model was simulated and initialized to a control state wherein all biomolecules attained control steady state values and was aligned to normal cell physiology that is non-tumorigenic. This non-transformed epithelial cell was triggered to transition into a neoplastic state by overlaying a trigger file on the network that introduced mutations in different oncogenes and/or tumor suppressors and other genetic and epigenetic changes that modulated the functional levels of intracellular molecules to align the system to different transformed states such as cell lines, patient profiles etc. The effect of a drug is introduced in the in silico model after determining the mechanism of action of each drug based on published research and extensive validation of the drug across simulation studies and correlation with experimental data.
