# Supplementary figures and images for: Novel anti-glioblastoma agents and therapeutic combinations identified from a collection of FDA approved drugs
Source: J Transl Med. 2014 Jan 17;12:13. doi: 10.1186/1479-5876-12-13 (PMC3898565; doi:10.1186/1479-5876-12-13)

## Slide 1
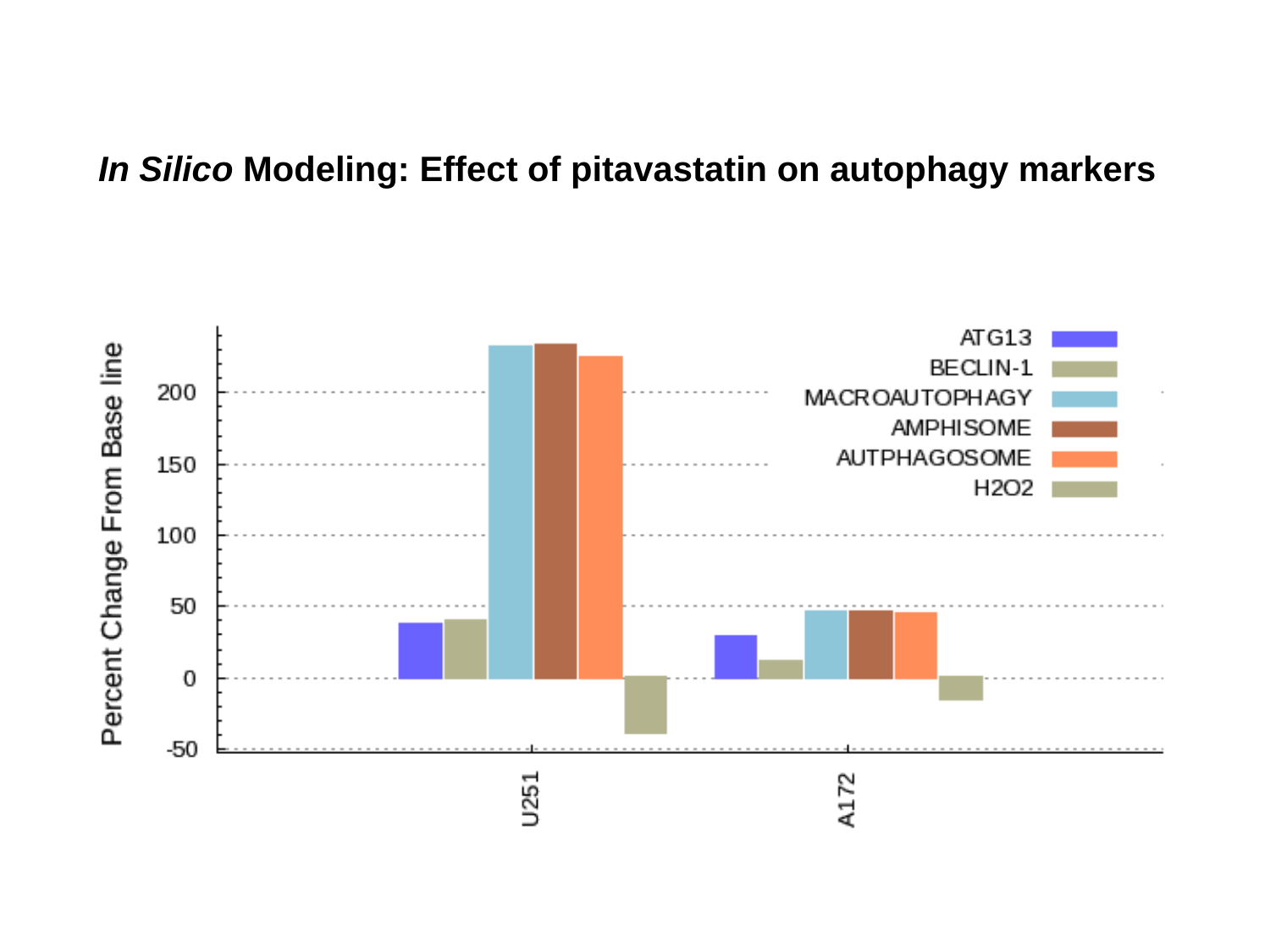

# In Silico Modeling: Effect of pitavastatin on autophagy markers

Supplement: Additional file 3: Figure S2 — In Silico modeling predicts pitavastatin-induced autophagy in GBM cell lines. We simulated the effect of pitavastatin on the virtual tumor cell that modeled GBM cell lines A172 and U251. Our simulation demonstrated increased expression of autophagy and related pathway markers in both these cell lines. [file 1479-5876-12-13-S3.ppt]
